# Envy, Politics, and Age

**DOI:** 10.3389/fpsyg.2013.00067

**Published:** 2013-03-07

**Authors:** Christine R. Harris, Nicole E. Henniger

**Affiliations:** ^1^Department of Psychology, University of CaliforniaSan Diego, CA, USA

**Keywords:** envy, emotion, age, political psychology, ideology, attitudes, emotion regulation

## Abstract

In the last 5 years, the phrase “politics of envy” has appeared more than 621 times in English-language newspapers, generally in opinion essays contending that political liberalism reflects and exploits feelings of envy. Oddly, this assertion has not been tested empirically. We did so with a large adult sample (*n* = 357). Participants completed a Dispositional Envy Scale and questions about political ideology, socioeconomic status, and age. Envy and age were moderately correlated; younger people reported greater envy. Political ideology and envy were weakly correlated; however, this relationship was not significant when controlling for age.

## Introduction

A vast number of writers have contended that liberal political doctrines reflect and appeal to enviousness on the part of voters. According to the *Newsbank* database, in the past 5 years there have been 621 references to the phrase “politics of envy” in US newspapers included in the database. In a typical reference of this kind, advocates of redistribution of wealth or taxation schemes designed to blunt economic inequality are denounced as exploiting this rather poorly regarded emotional state. An exact definition of envy is rarely offered in these writings, but what is clearly implied is that envy involves not only wanting what another has but also negative feelings and resentment over the greater success or material wealth of others, which fuels a desire to see some of that advantage taken away from those more fortunate. Such a definition is in keeping with how many psychologists define envy, although psychological research has also noted that envy does not only involve negative feelings toward others but often also has the consequence of making one feel bad about oneself (Smith and Kim, 2007).

The view of liberalism as a politics of envy has been advocated not only in numerous opinion essays, but also in several book-length treatments (e.g., de la Mora, 1987; Bandow, 1994) and is not a uniquely US phenomenon (e.g., a headline in the United Kingdom’s *Guardian* on 29 August 2012 was, “Nick Clegg wealth tax ‘the politics of envy,’ says senior Tory”). It is remarkable, therefore, that there appear to be no published empirical data on the question of whether political ideology in fact has the often-assumed relationship to people’s susceptibility to envy^1^.

In the present study, we explored this issue by using a published scale of enviousness for which validation data exist – the Dispositional Envy Scale (DES; Smith et al., 1999). Smith et al. showed that the scale correlated in a predictable fashion with several other measures of envy. They also found that high scorers on the DES were more prone to report envying a target person described as superior on a number of dimensions. Parks et al. (2002) have also provided elegant evidence for the criterion validity of the DES. Higher scorers on the scale were substantially more likely than lower scorers to choose noncooperative moves in a repeated-play prisoner’s dilemma game with a simulated opponent whose payoffs were arbitrarily set to be greater than the participant’s own payoffs. That is, individuals with high dispositional envy were more willing than other people to sacrifice some of their own winnings in order to reduce the winnings of an opponent who, for no apparent reason, was receiving richer payoffs than they were. The DES measure appears well suited for an initial examination of the putative relationship between enviousness and political ideology.

We also examine whether any relationship between political leanings and dispositional envy might be accounted for by differences in age or economic status. It is often-assumed that younger people lean more to the political left, and there is some evidence of this for the current younger generation (Levine et al., 2008). There is also emerging evidence that the experience of emotion changes with age. Older people appear to experience higher levels of positive affect and less negative affect than younger people, although this research has focused on more general experiences of emotion rather than specific emotions like envy (for reviews see Charles and Carstensen, 2007, 2009). Therefore, it is possible that if any empirical evidence for a correlation between political ideology and envy were to be revealed, it might arise because of differences across age groups in both ideology and propensity to experience envy. We test this possibility.

## Materials and Methods

Participants (*n* = 357) were recruited from two online sources: (1) Mechanical Turk and (2) our laboratory’s online research subject pool, which consists of a diverse panel of subjects of various ages and socioeconomic backgrounds. Subjects normally join the pool based upon referrals from search engines such as Google. The pool has been pre-screened in prior experiments for careful attention to instructions and conscientious performance.

In exchange for payment, participants completed an online survey. This method affords a high degree of anonymity, which may produce more candid responses to questions about socially undesirable attitudes such as envy (cf. Musch et al., 2001). The brief description that accompanied the advertisement for the study simply stated that it was about social and emotional experiences with no mention of envy or political ideology.

Participants provided key demographic information including gender, age, race/ethnicity, family income (assessed with 11 categories ranging from less than $10,000 to over $100,000), and education (assessed with six categories ranging from less than a high school degree to an advanced college degree). Participants were also asked if they were registered to vote, and if so, to indicate their political party. (Additional questions were asked that were not related to the current research topic and therefore are not reported here).

Political ideology was self-categorized on a five-point scale from (1) far-left, center-left, middle of the road, center-right, to (5) far-right. Finally, participants completed the DES (Smith et al., 1999). This scale consists of eight items including: (1) I feel envy every day. (2) The bitter truth is that I generally feel inferior to others. (3) Feelings of envy constantly torment me. (4) It is so frustrating to see some people succeed so easily. (5) No matter what I do, envy always plagues me. (6) I am troubled by my feelings of inadequacy. (7) It somehow does not seem fair that some people seem to have all the talent. (8) Frankly, the success of my neighbors makes me resent them. Participants respond to these items on a five-point scale from Strongly Disagree to Strongly Agree. The individual DES scores were summed for analyses. We also examine just items that focus on material wealth or success (items 4, 7, and 8) as it could be argued, as pointed out by a reviewer, that these most closely tap into the type of envy implied when the term “the politics of envy” is used.

## Results

The sample was 60% female, 62% Caucasian, and had a mean age of 34.4 years (range 18–70 years). The sample was diverse in terms of both income and education (see Table 1). Of the participants who were registered to vote, 43% (*n* = 128) reported being affiliated with the Democratic party and 20% (*n* = 59) with the Republican Party. The composite DES scale was computed by summing the individual response items (mean = 17.67, SD = 7.42, range 8–40, coefficient alpha = 0.898).

**Table 1:** **Distribution of participants’ education and income**.

Highest level of education	% of Participants (*n* = 357)
Some high school or less	1.4
High school diploma	13.4
Some college	38.4
Bachelor’s degree	31.4
Some graduate work	7.3
Advanced degree	8.1

**Income**	**(*n* = 351)**

Under $10,000	10.5
$10,000–19,999	10.8
$20,000–29,999	14.0
$30,000–39,999	12.8
$40,000–49,999	10.0
$50,000–59,999	8.3
$60,000–69,999	8.3
$70,000–79,999	6.0
$80,000–89,999	2.0
$90,000–99,999	3.4
More than $100,000	14.0

**Figure 1:**
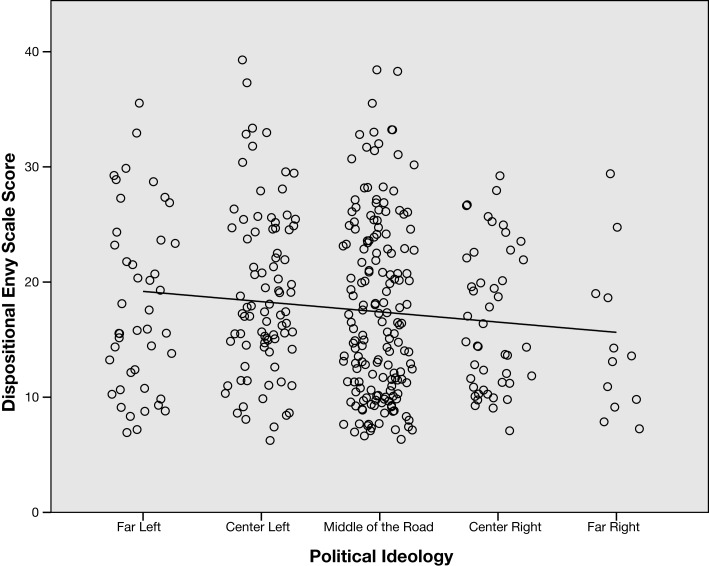
**Relationship between political ideology and dispositional envy**. Note: horizontal and vertical position has been jittered for better viewing.

**Figure 2:**
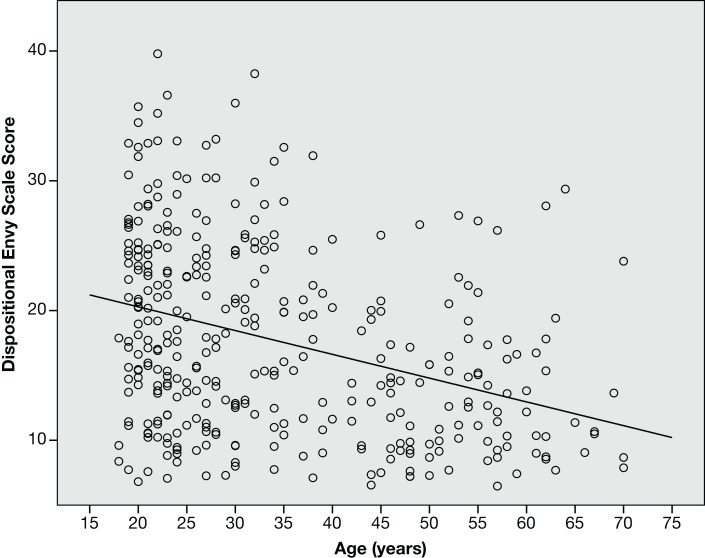
**Relationship between age and dispositional envy**. Note: vertical position has been jittered for better viewing.

Table 2 presents the correlations between dispositional envy, political ideology, and demographic variables. (The distribution of key variables differed significantly from normality according to Kolmogorov–Smirnov tests, therefore Spearman Rho was used to compute all correlations. Analyses using Pearson’s *r*, although not reported here, produced a strikingly similar pattern of results.) Dispositional envy was not related to income, education, or gender. There was a small but significant correlation between dispositional envy and political ideology; those who reported more left-wing ideology reported greater dispositional envy (see Figure 1). There was a stronger relationship between age and envy [*r*(349) = −0.337, *p* < 0.001]. As can be seen in Figure 2, younger people had higher DES scores.

**Table 2:** **Intercorrelations of dispositional envy, political views, and demographic variables**.

	DES	Political views	Age	Income	Education	Gender
Envy (DES)	–	−0.114*	−0.337***	−0.039	−0.036	0.045
Political views		–	0.122*	0.002	0.002	−0.092
Age			–	−0.097	0.128*	−0.038
Income				–	0.198***	−0.007
Education					–	−0.113*
Gender						–

*p < 0.05, ***p < 0.001.

*Gender: male = 1; female = 2*.

*Political views: far left = 1 to far right = 5*.

*Reported correlations are Spearman rho*.

Given the correlations between age, political ideology, and envy, one might wonder whether the relationship between envy and political ideology could be accounted for by the shared variance between age and political ideology. To examine this, we used a Partial Rank Correlation test to compute a partial correlation between political ideology and envy (controlling for age); which did not reveal a significant effect, *r*(346) = −0.073, *p* = 0.17. This indicates that there is no detectable relationship between envy and ideology apart from the variance shared with age. Of course, we cannot rule out the possibility that the tiny remaining trend (which would account for less than 1 percent of the variance) could be real.

We also examined whether just the three items from the DES that were related to envy over others’ success would reveal a stronger relationship to political ideology. The pattern of results was similar to, but weaker than, that found when the full DES was employed: the correlation between the reduced DES and political ideology was, *r*(355) = −0.10, *p* = 0.053. Using a Partial Rank Correlation test, the partial correlation between political ideology and the reduced DES controlling for age was also non-significant, *r*(352) = −0.068, *p* = 0.20.

Our final analysis assessed effects of party affiliation (being registered as Republican vs. Democratic). As expected, Democrats endorsed greater left-wing ideology than Republicans (*M* = 2.23, SD = 0.95 vs. *M* = 3.50, SD = 0.94, respectively): *t*(185) = −8.55, *p* < 0.001. However, while Democrats tended slightly toward more dispositional envy (*M* = 17.61, SD = 7.94) than Republicans (*M* = 15.98, SD = 6.79), this difference was not statistically significant: *t*(180) = 1.35, *p* = 0.18.

## Discussion

The results provide some evidence for the common claim that there is some relationship between political liberalism and enviousness. It appears, however, to be a weak relationship (at least as measured here). One could view this finding from two perspectives. On the one hand, a correlation of +0.11 accounts for less than 2% of the variance. Moreover, the correlation that does exist appears likely to be principally accounted for by youth being associated with liberalism and with envy. Given the small correlation, one might argue that it is unlikely that people would be able to detect such a weak relationship through informal observation (Jennings et al., 1982). Thus, one could suspect that the widespread belief that enviousness and liberalism are related probably mostly reflects commentators’ *a priori* theories rather than their informal empirical observations or that they have mistakenly attributed envy due to youth to envy due to political orientation.

On the other hand, one could argue that even if the true effect size of the correlation between envy and political liberalism is quite small, it might still be meaningful in the context of American politics. For example, as a reviewer points out, in presidential elections, the margins for the popular vote are often razor thin. One need only think of the Bush-Gore presidential election to see how close elections can be (where hanging chads could tip the balance). Therefore, even a small advantage provided by an emotion such as envy might be having important effects in the world.

The decline in envy scores with age – which does not appear to have been previously reported – is interesting in its own right. This result is consistent with other research that suggests that adult aging may lead to decreases in negative affect in general (Charles and Carstensen, 2007, 2009). The current study adds to this body of work by showing that a specific emotion, envy, also occurs less in older individuals. This raises the question of what might account for the age effect. One possibility, often implied in the literature, is that people get better at regulating their emotions as they age. For example, it has been suggested that such changes might be due to higher quality social relationships, decreased memory for or attention to negative events, greater avoidance of conflict and negative experiences, and altered appraisal of negative situations (see Charles and Carstensen, 2007, 2009). One also might wonder whether youth is associated with greater envy because the young have less and therefore have more to envy. One of our findings would argue against this possibility. There was a small tendency for younger people in our sample to have higher incomes than older people, and yet they still reported greater envy.

Another possibility is that the differences in envy across age groups are due to cohort effects rather than to developmental changes. It may be that socio-cultural differences across generations have led to younger people being more prone to envious reactions than their older counterparts, e.g., through greater emphasis on equity or differences in high self-esteem (see Gentile et al., 2010; Twenge and Foster, 2010, for suggestions of cohort effects in self-esteem). Whether any of these possibilities could account for our observed decline in enviousness with age would be worthy of future investigation.

### Limitations and future research

While this study challenges the assumption that there is a strong relationship between political liberalism and envy, it has a number of important limitations that should be acknowledged; some of these might be addressed in future research.

First, the Smith et al. (1999) measure of envy is fairly global, and is not specifically focused on envy of others based on their material possessions. It is conceivable that despite the present findings, envy focused on material possessions or wealth might be more strongly correlated with a more left-wing political orientation. To tentatively examine this possibility, we examined scores on just the three DES items that focus on success. Our results were no stronger when just this subset of envious reactions was examined. However, the possibility that materialistic envy might be more strongly correlated with political ideology may deserve further exploration with measures that are more tailored to specifically examining envy over what others own or have achieved.

A second fairly obvious limitation is that the DES relies on people admitting their own envy – through avowing rather unvarnished expressions of envy (e.g., “It is so frustrating to see some people succeed so easily”). As Silver and Sabini (1978) point out, envy “is felt to be nastier, more demeaning, less human than the other sins” (see also Epstein, 2003). Thus, it seems plausible that some people might disavow the emotion even if they experience it frequently (a point acknowledged by Smith et al., 1999). However, our participants varied greatly in their DES scores, indicating that they were using a wide range of the scale (this can also be seen in Figure 1). Also, even if the DES seriously underestimates the enviousness of our sample, one would still expect in a large sample to detect a unique relationship between relative levels of envy and political liberalism, if any strong relationship existed. Moreover, the current work was capable of revealing a moderate correlation between age and disposition toward envy. In future research, it would be interesting to try to devise more indirect measures of envy to get around this limitation.

Finally, a question that one might reasonably ask, in light of these results, is *why* there is not a stronger relationship between envy and political ideology. After all, someone with an envious disposition will surely notice the great wealth of some members of his or her society, so why would they not be inclined to envy it? And why should that in turn *not* powerfully drive them to embrace an ideology that labels their comparative disadvantage as problematic and seeks (at least in small measure) to mitigate it?^2^

One possible answer is that someone who adheres to a political philosophy that views extremes of wealth as immoral or at least undesirable might not subjectively experience or label their negative reactions to wealthier people as “envy.” Instead, they might label these feelings as legitimate “resentment” or “disapproval” – moral attitudes that they might categorize as entirely separate from the private vice of envy (Feather, 1999; Feather and Sherman, 2002). As Smith (1991) notes, “another’s legitimate advantage (is often) construed by the envying person as illegitimate, so that envious feelings come across to oneself and to others as resentment or righteous indignation rather than envy.” (p. 81; see also Rawls, 1971). Given this transformation, for those holding liberal ideologies, negative affective reactions to wealthier individuals may typically not enter into their responses to DES questions. Future research could examine how political ideology is related to resentment over injuries and slights in various realms, including both those having a political dimension and those lacking it.

It is also possible that the weak link between political ideology and envy is due to our single measure of political orientation, which asked people to place themselves on a left-right continuum (although we also did not find a significant relationship between party affiliation and envy). Political ideology is complex and it may be that more nuanced measures (such as how fiscally conservative or socially liberal one is) might reveal stronger relationships between envy and political attitudes.

In summary, the data described here found weak support for the widespread contention that envy and political liberalism are linked, and suggested that what linkage exists is principally due to the tendency of envy to weaken with age (with liberalism weakening somewhat over the same period). However, the relationship between political ideology and sentiments such as envy and resentment is potentially multifaceted and complex, so the topic deserves further empirical study. It is hoped the present article may help spark interest in this potentially rich area of investigation.

## Conflict of Interest Statement

The authors declare that the research was conducted in the absence of any commercial or financial relationships that could be construed as a potential conflict of interest.

## Footnotes

^1^The only opposition to a linkage of envy and liberalism that we are familiar with appears in a discussion by Neu (1980, p. 435), who merely objects to drawing any policy conclusions from the linkage, saying “that envy may be one reason for demanding equality does not mean that demands for equality are unjustified. For one thing, envy may be justified.”

^2^Note that this does not assume that political attitudes necessary reflect economic self-interest; cf. Sears et al. (1980) for evidence suggesting that symbolic factors are often more potent than concrete self-interest in determining political preferences.
